# Infratemporal Fossa Approach with Preservation of the Posterior Bony Wall of External Auditory Canal: Case Series and the Outcome

**DOI:** 10.3390/jcm14155294

**Published:** 2025-07-26

**Authors:** Hye Ah Joo, Na-Kyum Park, Jong Woo Chung

**Affiliations:** 1 Department of Otorhinolaryngology, Dongtan Sacred Heart Hospital, Hallym University College of Medicine, Hwaseong 18450, Republic of Korea; hyeah_j@hallym.or.kr; 2 Department of Otorhinolaryngology-Head and Neck Surgery, Asan Medical Center, University of Ulsan College of Medicine, Seoul 05505, Republic of Korea; d210778@amc.seoul.kr

**Keywords:** infratemporal fossa approach, paraganglioma, conductive hearing loss, facial nerve, external auditory canal

## Abstract

**Objective:** To evaluate the outcomes of a modified infratemporal fossa approach (ITFA) that preserves the posterior external auditory canal (EAC) in patients with tumors in the infratemporal fossa and skull base, focusing on postoperative hearing and facial nerve function. **Methods:** This retrospective study included nine patients who underwent ITFA with posterior EAC preservation for tumor removal while minimizing facial nerve rerouting. All surgeries were performed by a single surgeon. Preoperative and postoperative hearing levels, facial nerve function, tumor characteristics, and surgical outcomes were analyzed. Air-bone gaps (ABG) were assessed using pure tone audiometry, and facial nerve function was assessed using the House–Brackmann grading system. **Results:** The cohort consisted of eight female patients and one male patient, with a mean tumor size of 3.0 cm. Surgical outcomes were promising, with no statistically significant increase in postoperative ABG and well-preserved facial nerve function. Only one patient developed postoperative grade II facial palsy. A residual tumor was identified in one case with extensive meningioma, which has remained stable, and no recurrence or regrowth was noted during the follow-up period (mean: 3.7 years). The modified approach minimized complications related to conductive hearing loss and facial nerve dysfunction. **Conclusions:** The modified ITFA with posterior EAC preservation provides a promising alternative to conventional ITFA for managing deep-seated tumors. It preserves both hearing and facial nerve function while ensuring adequate tumor resection.

## 1. Introduction

The infratemporal fossa is an anatomically complex area bounded superiorly by the greater wing of the sphenoid, medially by the lateral pterygoid plate, laterally by the mandibular ramus, anteriorly by the posterior border of the maxillary sinus, and posteriorly by the carotid sheath [1]. It contains the maxillary artery, the mandibular branch of the trigeminal nerve, the pterygoid muscles, and the pterygoid venous plexus. The intricate anatomical layout of this region necessitates a precise and carefully planned surgical approach to minimize the risk of damage to the critical neurovascular structures that traverse this area.

The infratemporal fossa approach (ITFA), first described by Fisch in the late 20th century, revolutionized the management of deep-seated skull base lesions by providing extensive exposure of the infratemporal fossa while allowing for the safe resection of tumors and other pathological entities [2]. This pivotal surgical technique grants access to deep-seated lesions within the infratemporal fossa, jugular foramen, skull base, and cervical carotid area. It is particularly effective for managing complex tumors and other pathological entities that are challenging to approach owing to their intricate anatomical locations. The primary indication for employing ITFA is the removal of glomus tumors in the jugular foramen; however, this approach is versatile and can also be utilized to excise other pathologies, such as cholesteatomas, schwannomas, inflammatory lesions, and various malignant tumors [3,4].

A significant aspect of the ITFA is the rerouting of the facial nerve [2]. Owing to its complex course through the temporal bone, the facial nerve is a critical structure at risk during surgeries involving the skull base and the infratemporal fossa. In the conventional ITFA, the facial nerve is mobilized from its original anatomical position to enhance surgical exposure and facilitate the safe removal of lesions. However, this extensive rerouting can lead to postoperative complications, most notably facial nerve damage. Patients may experience facial asymmetry and varying degrees of facial nerve palsy, significantly impacting their quality of life [5]. In cases where limited rerouting of the facial nerve is employed, postoperative facial function has been shown to be preserved in the long-term follow-up [6].

Another critical aspect of the ITFA is the removal of the posterior bony wall of the external auditory canal (EAC). This step is necessary to achieve adequate exposure and access to the lesion. Following the removal of the bony wall, reconstruction of the middle ear becomes essential. Reconstruction techniques involve the use of fascia or the obliteration of the middle ear cavity with fat, followed by the closure of the EAC. Despite these efforts, patients typically experience postoperative big conductive hearing loss owing to the disruption of the auditory canal and middle ear structures [7].

Glomus tumors, also known as paragangliomas, are the most common tumors of the jugular foramen [8]. They originate from neural crest-derived chemoreceptor cells of the endocrine system [4,9]. While these tumors are typically benign, they can exhibit locally aggressive behavior, making surgical resection the preferred treatment modality. The ITFA has traditionally been the approach of choice for these tumors owing to the extensive exposure it provides [3,7]. However, concerns regarding the impact of the conventional approach on facial nerve function and hearing have prompted the exploration of modified techniques to enhance postoperative outcomes [10].

Given these significant postoperative morbidities associated with the conventional ITFA, there is a pressing need to refine the technique to improve patient outcomes [11]. One such refinement involves preserving the posterior EAC during the approach. By maintaining the integrity of the EAC with limited rerouting of the facial nerve, it is hypothesized that both facial nerve function and auditory outcomes could be significantly improved [6,12]. This study aimed to evaluate the postoperative results of a modified ITFA technique that preserves the posterior EAC in patients with tumors in the jugular foramen.

## 2. Materials and Methods

This study is a retrospective analysis of patients who underwent the ITFA with preservation of the posterior EAC for the removal of tumors in the jugular foramen at a tertiary care center. We included patients diagnosed with jugular foramen tumors who underwent surgery between January 2013 and December 2023. Patients with tumors confined solely to the middle ear were excluded, as these cases were suitable for a transcanal microscopic or endoscopic approach and did not require ITFA. All procedures were performed by a single surgeon (J.W.C.). Patients are typically followed at 2–4 weeks postoperatively, at 3 months, and then annually thereafter with clinical examinations and imaging as needed.

Data were collected retrospectively from medical records and included preoperative, intraoperative, and postoperative information. The following variables were analyzed: age, sex, tumor site, presenting symptoms, tumor size, preoperative and postoperative pure tone audiometry (PTA), evidence of facial palsy, follow-up duration, and the presence of residual tumor or recurrence.

Hearing levels were assessed both preoperatively and postoperatively using the average of the four-frequency pure tone thresholds at 0.5, 1, 2, and 4 kHz [13]. Differences between preoperative and postoperative air-bone gaps (ABGs) were categorized based on a 10 dB threshold: a worsening of 10 dB HL or more was classified as “worse”, an improvement of 10 dB HL or more was categorized as “better”, and changes within that range were considered “same.” Facial nerve function was evaluated preoperatively and postoperatively using the House–Brackmann grading system, which ranges from Grade I (normal function) to Grade VI (complete paralysis) [14].

Descriptive statistics were used to summarize patient demographics, tumor characteristics, and surgical details. Continuous variables were reported as means ± standard deviations, while categorical variables were presented as frequencies and percentages. Preoperative and postoperative hearing levels and facial nerve function were compared using the Wilcoxon signed-rank test to assess significant differences following surgery. Statistical significance was defined as *p* < 0.05.

The study was conducted in accordance with the principles of the Declaration of Helsinki. Ethical approval was obtained from the Institutional Review Board of Asan Medical Center (2024-0121). Owing to the retrospective nature of the study, the requirement for obtaining informed consent was waived.

## 3. Results

The study included nine patients (eight female, one male). The demographics and clinical features of these patients are summarized in Table 1. Clinical presentations varied, with the main symptoms being hearing loss and tinnitus. The mean age at the time of surgery was 48.2 (range: 30–61) years. The mean tumor size was 3 cm (median: 2.1 cm, range 1.5–9.1 cm), with tumor locations involving the right ear in five cases and the left ear in four cases. Histological examination revealed seven paragangliomas, one meningioma, and one endolymphatic sac tumor. The average follow-up period was 4.2 ± 3.1 years. During the follow-up periods, no recurrent mass was identified in all patients.

In the eight patients with paraganglioma and one endolymphatic sac tumor, the main feeding vessels were the ascending pharyngeal artery and occipital artery. Minor feeders included the anterior tympanic and caroticotympanic arteries. Preoperative neurologic examinations revealed that all patients had intact lower cranial nerve function except for hearing loss, and no other cranial nerve symptoms were reported.

To illustrate the application of the modified ITFA technique with preservation of the posterior bony wall of the EAC, two surgical cases are described in detail as illustrative examples.

### 3.1. Case 1 (Patient 5)

**Figure 1:**
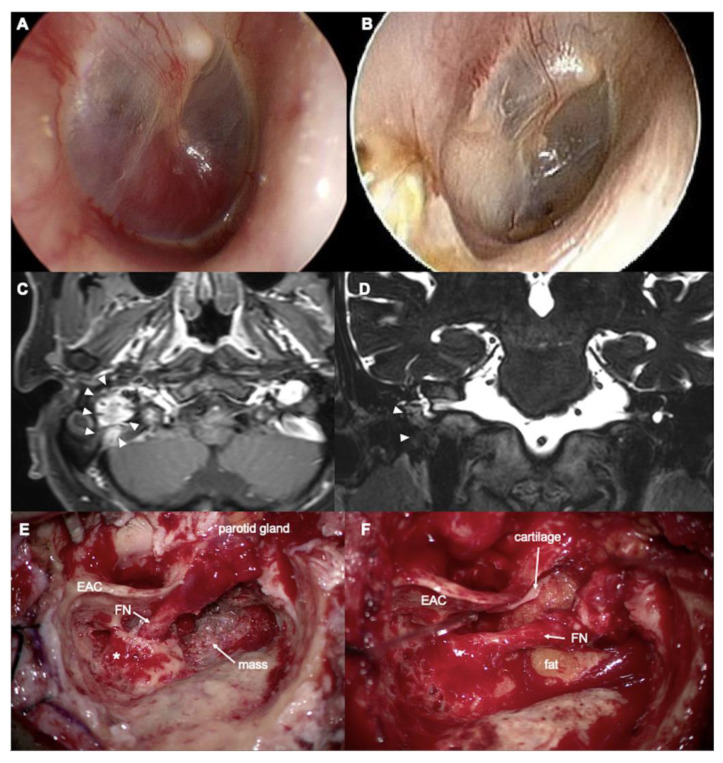
A 61-year-old man with paraganglioma. (**A**) Preoperative and (**B**) 3-year postoperative tympanic membrane. (**C**) Axial T1- and (**D**) coronal T2-weighted MRI. (**E**,**F**) Intraoperative findings. arrowheads: mass, EAC: external auditory canal, FN: facial nerve, * lateral semicircular canal.

A 61-year-old man presented with right-sided hearing loss, accompanied by pulsatile tinnitus during head movement and a sensation of ear fullness for two years. Tympanic endoscopy revealed a pulsatile reddish mass behind the right tympanic membrane (Figure 1A). Temporal bone computed tomography (CT) and magnetic resonance imaging (MRI) (Figure 1C,D) demonstrated a 2.3 cm well-enhancing, hypervascular soft tissue mass in the middle ear and jugular foramen, highly suggestive of a glomus jugulare and tympanicum. The inferomedial bony wall of the EAC was eroded. The air conduction pure tone average in the right ear was 45 dB HL, with an ABG of 4 dB HL. No facial palsy was noted preoperatively. Preoperative embolization of the right occipital artery and postauricular artery was performed.

**Figure 2:**
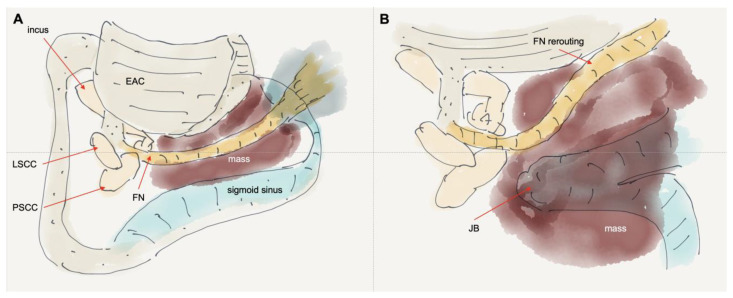
Illustration of the surgical process in Case 1. (**A**) Canal wall-up mastoidectomy with FN skeletonized. (**B**) FN rerouted anteriorly (short rerouting) to expose the mass in hypotympanum and jugular foramen. EAC: external auditory canal; LSCC: lateral semicircular canal; PSCC: posterior semicircular canal; FN: facial nerve; JB: jugular bulb.

Surgery was performed using a modified IFTA technique, preserving the posterior EAC. After a complete canal wall-up mastoidectomy and posterior tympanotomy, the facial recess was widened inferiorly by removing the inferior portion of the bony EAC. A vascular mass was identified in the hypotympanum and jugular fossa. The facial nerve was skeletonized and rerouted anteriorly from the pyramidal segment to the parotid segment (short rerouting) (Figure 1E). The entire mass was removed from the hypotympanum, mastoid cavity, and jugular fossa. The tumor was adhered to the jugular bulb without internal invasion. The adhered mass was carefully removed, and bleeding from the jugular bulb was controlled using Gelfoam. The inferior EAC bony defect was covered by cartilage and supported by fat obliteration of the mastoid cavity (Figure 1F). Postoperative air conduction PTA was 44 dB HL with an ABG of 0 dB at the 3-year follow-up. The latest follow-up MRI, conducted 3.5 years after surgery, showed no recurrence of the tumor. The patient had an intact right tympanic membrane and EAC (Figure 1B). Figure 2 shows an illustration of the surgical process.

### 3.2. Case 2 (Patient 3)

**Figure 3:**
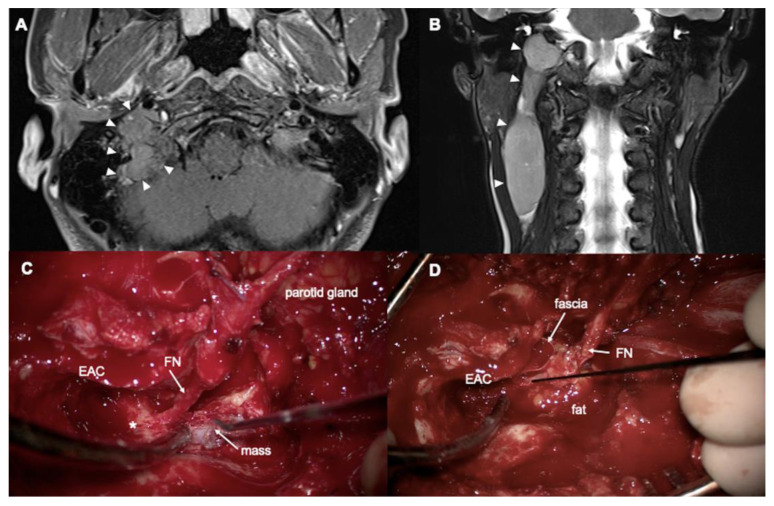
A 42-year-old woman with angioinvasive paraganglioma. (**A**) Axial T1- and (**B**) coronal T2-weighted MRI show a mass from jugular foramen to right neck (arrowheads). (**C**,**D**) Intraoperative findings. arrowheads: mass, EAC: external auditory canal, FN: facial nerve, * lateral semicircular canal.

A 42-year-old woman presented with right-sided tinnitus, otalgia, and a palpable neck mass. Tympanic endoscopy revealed a clear right tympanic membrane. CT and MRI demonstrated an approximately 9.1 cm sausage-like intravascular mass in the right internal jugular vein, extending from the jugular foramen to the level III neck, consistent with a diagnosis of paraganglioma (Figure 3A,B). Preoperatively, the patient exhibited normal hearing without an ABG and showed no signs of facial palsy. Tumor embolization was performed preoperatively via the right ascending pharyngeal artery, occipital artery, and posterior auricular artery.

After performing a complete canal wall-up mastoidectomy and posterior tympanotomy while preserving the posterior EAC, the mastoid segment of the facial nerve was fully exposed through drilling. Following partial superficial parotidectomy, neck dissection, and mastoid tip removal, the facial nerve was partially rerouted anteriorly (“short” rerouting). The entire tumor, extending from the jugular bulb to the internal jugular vein, was then identified (Figure 3C). The jugular bulb was opened, and bleeding control was achieved at the inferior petrosal sinus. After ligating the right internal jugular vein, the tumor was removed while maintaining bleeding control throughout the procedure. The partially rerouted facial nerve was subsequently repositioned, and the mastoid cavity was obliterated with an abdominal fat graft (Figure 3D). Histological examination confirmed the diagnosis of angioinvasive paraganglioma. Owing to the tumor’s extensive intravascular growth, the patient underwent adjuvant radiotherapy. Postoperative hearing measurements showed an ABG of 8.75 dB HL and an air conduction PTA of 22.5 dB HL. A follow-up MRI at 2.5 years after surgery showed no evidence of tumor recurrence.

Table 2 summarizes the changes in hearing thresholds and facial nerve function following the application of the modified ITFA technique. Regarding hearing thresholds, two patients experienced deafness owing to the use of a combined translabyrinthine approach to remove the mass in the internal auditory canal. In contrast, seven patients with paraganglioma (glomus jugulare) showed preserved postoperative hearing levels.

Postoperatively, one patient (Patient 2) showed a notable improvement in ABG, with a reduction of 17.5 dB HL, while the other four patients (whose ABG was measurable) demonstrated no significant changes, with differences remaining within 10 dB HL. However, the patient with a meningioma (Patient 4) and the patient with an endolymphatic sac tumor (Patient 6) were determined to be deaf postoperatively.

Postoperative facial palsy was observed in only one patient (Patient 1), who presented with House–Brackmann grade II at the 1-year follow-up and the last follow-up (3.3 years). All other patients exhibited normal facial function at the 1-year postoperative evaluation. Overall, no statistically significant changes in facial nerve function were observed following the modified ITFA technique (*p* = 0.317).

The mean follow-up period was 3.7 (range: 1.0–11.7) years, during which residual tumor was observed in one case (14.3%). This patient (Patient 4) had a petroclival meningioma involving the bilateral skull base and had already undergone two tumor removal surgeries on each side, performed by a neurosurgeon. The remnant tumor involving the right middle ear and jugular foramen was additionally removed in our department using the modified ITFA. A small mass medial to the right jugular foramen remained, as it extended into the retroclival region. Postoperatively, the patient underwent radiation therapy for the residual mass. The residual tumor in the bilateral retroclival region and medial side to the right jugular foramen is being closely monitored, with no change in size observed during regular follow-up MRIs. The other patients were under close observation at the time of this writing, and no recurrences or other postoperative complications were observed in any case.

## 4. Discussion

The preservation of the posterior EAC during ITFA is proposed to offer several potential benefits. First, it is anticipated that maintaining the structural integrity of the EAC will reduce the degree of conductive hearing loss by preserving the natural anatomy and function of the auditory canal. Additionally, by avoiding the extensive rerouting of the facial nerve, the risk of postoperative facial nerve damage and associated complications may be minimized. This refined approach has the potential to significantly improve the quality of life of patients undergoing surgery for glomus tumors in the jugular foramen.

To date, limited research has been conducted on the outcomes of the ITFA with preservation of the posterior EAC. This study aimed to address this gap by systematically evaluating postoperative hearing and facial nerve function in patients who have undergone this modified surgical approach. The primary endpoints included the degree of postoperative conductive hearing loss, assessed through PTA, and the extent of facial nerve function impairment, evaluated using standardized facial nerve grading systems. Secondary endpoints included overall surgical morbidity, completeness of tumor resection, and recurrence of the lesion.

A total of seven surgical cases performed by a single surgeon were reviewed. The refined ITFA surgical technique demonstrated promising outcomes, with no statistically significant increase in postoperative ABG and well-preserved overall facial nerve function. Notably, one patient exhibited a significant improvement in ABG owing to the resolution of a conductive hearing disturbance caused by a middle ear tumor. These findings suggest that this modified surgical technique can effectively remove tumors while minimizing postoperative complications, thereby enhancing patients’ overall quality of life. Additionally, although residual tumor was identified in one patient with a primarily extensive meningioma on postoperative imaging, there were no instances of recurrence or regrowth during the follow-up period. This suggests that the preservation of the posterior EAC does not compromise surgical efficacy, alleviating concerns that a less extensive approach might lead to incomplete tumor removal.

In conventional IFTA, the tympanic membrane and EAC are typically removed, with the meatus overclosed, resulting in maximal conductive hearing loss [2]. To address this, Gold et al. proposed a technique for EAC reconstruction using hydroxyapatite cement. This method involves replacing the canal wall segment and securing it in place with hydroxyapatite cement following tumor resection [15]. In their study, patients who underwent successful EAC reconstruction demonstrated a mean difference of 7.5 dB HL in postoperative pure tone averages compared to preoperative values. As analyzed in our case series, a modified ITFA technique that preserves the posterior EAC maintains the anatomical integrity of the auditory canal and minimizes disruption to sound conduction mechanisms.

Although the conventional IFTA provides excellent exposure to the surrounding anatomy, complete anterior mobilization of the facial nerve from its first genu often results in facial palsy [2]. This outcome arises from both devascularization of the nerve and the maneuvers required to free the fibrous connections between the nerve and the fallopian canal. Llorente et al. found that in patients who underwent ITFA, facial nerve function was significantly better in the no-rerouting group compared to the rerouting group [12]. Furthermore, some studies have reported that “short” anterior rerouting (mobilization of the facial nerve to the second genu) results in the least facial nerve dysfunction, whereas “long” rerouting (mobilization to the geniculate ganglion) is associated with worse outcomes [6,16]. These findings highlight that the extent of facial nerve dysfunction is proportional to the length of nerve mobilized. Yi et al. also reported that when short rerouting is appropriately selected based on tumor and patient characteristics, the rate of complete tumor resection is higher, and the mean operation time does not significantly affect the postoperative recovery of facial function [6]. For the removal of glomus jugulare tumors between 2008 and 2015, we performed the traditional ITFA—comprising canal wall down mastoidectomy, long facial nerve rerouting, and obliteration of the mastoid cavity with fat—in ten patients with tumors involving the jugular foramen and middle ear. The average tumor size was 2.7 ± 0.64 cm, comparable to the current series. Facial nerve rerouting was short in two cases and long in eight. At one year postoperatively, facial nerve function was HB grade I in five patients, grade II in two, and grade III in three. All patients experienced large conductive hearing loss postoperatively. As shown in the results of this study, our refined ITFA technique minimizes the need for extensive facial nerve manipulation by preserving the anatomical course of the facial nerve, thereby reducing the risk of postoperative facial nerve dysfunction.

Despite the promising results, our study has several limitations. The mean size of the tumors in this series was 3.0 cm, suggesting that this technique may be most feasible for smaller tumors. The absence of a control group of patients who underwent conventional ITFA without EAC preservation also limits the ability to assess the specific benefits of preserving the posterior EAC wall, particularly in cases involving larger tumors affecting the carotid artery or deep petrous bone. Although the follow-up period (mean: 4.2 years) was sufficient to evaluate immediate and short-term outcomes, it may not be adequate to fully determine long-term recurrence rates or late-onset complications. The small sample size, single-center design, and specific selection criteria limit the generalizability of our findings. These factors may introduce selection bias and reduce external validity.

Future studies should focus on prospective, multicenter designs with larger sample sizes and control groups, as well as incorporate extended follow-up periods, to provide a more comprehensive evaluation of the long-term efficacy and safety of the modified approach.

## 5. Conclusions

In our retrospective analysis of nine patients, the IFTA with preservation of the posterior EAC achieved adequate tumor resection while preserving postoperative hearing function and facial nerve integrity, ultimately enhancing the quality of life for patients by minimizing postoperative complications. The favorable outcomes observed in this study support further investigation and validation through larger, prospective studies with control groups. While the modified ITFA technique demonstrates promising surgical and functional outcomes, these findings must be interpreted with caution due to the limited sample size. Further validation through larger, prospective studies is necessary.

## Figures and Tables

**Table 1:** Demographics and clinical features of seven patients who underwent the modified infratemporal fossa approach.

Patient	Age (Years)	Sex	Site	Presenting Symptoms	Radiologic Tumor Size (cm)	Pathology	Residual	Further Treatment	Follow-Up Period (Year)
1	55	F	R	Tinnitus	1.8	Paraganglioma	-	None	3.3
2	46	F	L	Hearing loss, tinnitus	1.8	Paraganglioma	-	None	3.0
3	42	F	R	Tinnitus, otalgia	9.1	Angioinvasive paraganglioma	-	RTx	2.2
4	42	F	R	Hearing loss, tinnitus	3.7	Meningioma	+	None	4.0
5	61	M	R	Hearing loss	2.3	Paraganglioma	-	None	4.4
6	30	F	R	Hearing loss, tinnitus	3.0	Papillary endolymphatic sac tumor	-	GKRS	12.2
7	60	F	L	Hearing loss, tinnitus	1.5	Paraganglioma	-	None	5.2
8	58	F	L	Hearing loss, tinnitus	2.1	Paraganglioma	-	None	1.6
9	40	F	L	Tinnitus	1.7	Paraganglioma	-	None	1.5
Average	48.2 ± 10.2				3.0 ± 2.3				4.2 ± 3.1

Averages are presented as mean ± standard deviation. F, female; M, male; R, right; L, left; GKRS, gamma knife radiosurgery; RTx, radiotherapy.

**Table 2:** Changes in hearing thresholds and facial nerve function following the surgery.

Patient	Hearing Threshold							Facial Nerve Function	
	Preop Mean PTA (dB HL)			Postop Mean PTA (dB HL)			Change in ABG *	HB Grade	
	BC	AC	ABG	BC	AC	ABG		Preop	Postop
1	21.25	22.50	1.25	16.25	22.50	6.25	same	I	II
2	23.75	53.75	30.00	7.50	20.00	12.50	better	I	I
3	1.25	1.25	0.00	13.75	22.50	8.75	same	I	I
4	42.50	78.75	36.25	deaf	deaf	N/A	worse	I	I
5	41.25	46.25	5.00	41.25	45.00	3.75	same	I	I
6	63.75	83.75	20.00	deaf	deaf	N/A	worse	I	I
7	41.25	43.75	2.50	55.00	58.75	3.75	same	I	I
8	55.9	59.1	3.2	50.0	57.5	7.5	same	I	I
9	19.2	22.5	3.3	10.0	13.5	3.5	same	I	I

ABG, air-bone gap; AC, air conduction; BC, bone conduction; HB grade, House–Brackmann grade; PTA, pure tone audiometry; Preop, preoperative; Postop, postoperative; N/A, not applicable. * Difference between changes in ABG was categorized as worse, same, and better, as described in the Section 2.

## Data Availability

The datasets generated during and/or analyzed during the current study are available from the corresponding author on reasonable request.
